# Assessing the Incidence, Persistence and Clearance of Oral HPV Detection Among Metropolitan and Non-Metropolitan Indigenous Australian Adults

**DOI:** 10.3390/v18101054

**Published:** 2026-09-23

**Authors:** Nicolas E. Reid, Xiangqun Ju, Joanne Hedges, Sneha Sethi, Lisa M. Jamieson

**Affiliations:** Australian Research Centre for Population Oral Health, School of Dentistry, College of Health, Adelaide University, Level 4, 50 Rundle Mall Plaza, Adelaide, SA 5000, Australia; nicolas.reid@adelaide.edu.au (N.E.R.); joanne.hedges@adelaide.edu.au (J.H.); sneha.sethi@adelaide.edu.au (S.S.); lisa.jamieson@adelaide.edu.au (L.M.J.)

**Keywords:** human papillomavirus (HPV), oropharyngeal cancer (OPC), Aboriginal and Torres Strait Islander, longitudinal cohort study

## Abstract

Background: Human papillomavirus (HPV) infects skin and mucous membranes, and high-risk types are associated with oropharyngeal and other cancers. Longitudinal evidence on oral HPV among Aboriginal and Torres Strait Islander peoples remains limited, particularly across geographic settings. Methods: A longitudinal cohort study was conducted among 1011 Indigenous Australian adults in South Australia recruited through Aboriginal Community Controlled Health Organisations. Participants provided saliva samples and completed questionnaires at baseline (2018–2019) and at 12- and 24-month follow-up. Oral HPV was detected using PCR-based assays. Incidence, persistence, and clearance were estimated, and multivariable regression examined associations with behavioural, sexual health, and socioeconomic factors. Results: Oral HPV dynamics differed between metropolitan and non-metropolitan participants. Approximately one-quarter developed an incident infection, with geographic variation in persistence and clearance. Smoking, sexual behaviours, and socioeconomic factors were associated with HPV outcomes. Older age was associated with persistence among non-metropolitan participants, while smoking was associated with higher incidence among metropolitan participants. Conclusions: Oral HPV detection dynamics varied by geographic setting. Culturally appropriate, targeted public health strategies addressing behavioural and social determinants are needed to reduce HPV-related disease burden.

## 1. Introduction

Human papillomavirus (HPV) is a diverse group of DNA viruses that infect epithelial tissues, including skin and mucous membranes. Over 200 different types of HPV have been identified, which are classified into high-risk HPV (hrHPV) and low-risk HPV (lrHPV) categories based on their association with the development of cancer [1]. High-risk HPV types, such as HPV-16 and HPV-18, are strongly associated with the development of cervical, anal, vulvar, vaginal, penile, and oropharyngeal cancers. These high-risk types are more likely to persist and progress to cancer compared to low-risk types [2].

Low-risk HPV types, such as HPV-6 and HPV-11, are commonly associated with benign conditions like genital warts and respiratory papillomatosis. Although these types are not typically linked with cancer, they can cause significant morbidity and psychological distress [3]. HPV types 13 and 32 are associated with focal epithelial hyperplasia (Heck’s disease), a rare HPV-related oral condition characterised by multiple papular lesions of the oral mucosa. Although it occurs more commonly in some Indigenous populations, its epidemiology should not be considered representative of the broader epidemiology of oral HPV detection [4].

The oral cavity and oropharynx are anatomically distinct regions and should be considered separately when interpreting HPV-related disease. Oropharyngeal cancer (OPC), which commonly involves the tonsils, base of the tongue, and soft palate, is strongly associated with high-risk human papillomavirus (hrHPV), particularly HPV16. However, detection of HPV DNA in saliva does not necessarily indicate infection at a specific anatomical site, nor does it imply the presence of cancer or a direct risk of oral cavity cancer. Most oral HPV detections do not progress to malignancy, and the oncogenic relevance of HPV is more clearly established for the oropharynx than for the oral cavity.

HPV is implicated in a substantial proportion of OPC cases, with some studies estimating that up to 70% are HPV-related. In Australia, the incidence of HPV-related OPC has increased, particularly among men, with the age-standardised incidence rate rising from 1.7 per 100,000 population in 2006 to 3.6 per 100,000 in 2015 [5,6,7,8,9,10]. HPV-related OPC is more common in men and is associated with sexual behaviours that may increase exposure to oral HPV. Although the burden of HPV-related OPC is greater among men, understanding patterns of oral HPV detection remains relevant in both men and women. Treatment commonly involves surgery, radiotherapy, chemotherapy, or combinations of these approaches and may substantially affect quality of life [7,11].

Aboriginal and Torres Strait Islander peoples experience higher rates of head and neck cancers and HPV-related conditions compared to non-Indigenous populations [12]. This disparity is attributed to various factors, including socioeconomic inequalities and limited access to healthcare services. Given these disparities, it is crucial to understand the demographic characteristics, including risk factors and determinants of HPV infections in these populations, to develop targeted public health interventions [13,14,15,16].

Classifying participants as metropolitan or non-metropolitan provides important insights into geographic differences in demographic characteristics, healthcare access, socioeconomic conditions, health behaviours, and environmental exposures. Metropolitan areas generally have higher population density, greater access to healthcare services, and different social contexts than non-metropolitan areas. These factors may influence the incidence, persistence, and clearance of oral HPV detection. Understanding such differences can support the development of public health interventions tailored to the needs of each population [16].

This study investigated longitudinal patterns of oral HPV detection, including incidence, persistence, and clearance, among Indigenous Australian adults living in metropolitan and non-metropolitan areas. The individual participant was the unit of analysis, with oral HPV detection assessed at baseline, 12 months, and 24 months. We also examined associations of incident and persistent oral HPV detection with sociodemographic characteristics, smoking and other health-related behaviours, sexual behaviours, and socioeconomic factors. A priori, we hypothesised that these factors would be associated with incident and persistent oral HPV detection and that the magnitude and pattern of associations might differ by residential location.

## 2. Materials and Methods

This study is reported according to STROBE (Strengthening the Reporting of Observational Studies in Epidemiology) guidelines.

### 2.1. Study Design and Participants

Eligible participants were Indigenous, over the age of 18 and residing in either metropolitan or rural South Australia were recruited using the methodology described in a previous publication [17]. This longitudinal cohort study collected baseline data between February 2018 and January 2019 from a large convenience sample of Indigenous Australian adults in South Australia aged 18+ years and older (n = 1011), recruited through Aboriginal Community Controlled Health Organisations (ACCHOs). Follow-up data were collected were at 12 months (February 2019 and January 2020; n = 764) and 24 months (February 2020 and January 2021; n = 803).

### 2.2. Ethics

Ethical approval for the study was obtained from the University of Adelaide Human Research Ethics Committee (H-2016-246) and the Aboriginal Health Council of South Australia (04-17-729). All participants willingly provided written informed consent.

### 2.3. Data Collection

Data collection was conducted by trained field research officers, with the support and oversight of a senior Indigenous research officer to ensure culturally sensitive and accurate data acquisition. Participants provided detailed information on their demographic characteristics, health behaviours, and sexual history, contributing to a comprehensive dataset for subsequent analysis. Saliva samples were collected for hrHPV testing, with follow-up samples obtained at 12 months (n = 764) and 24 months (n = 803) to monitor infection dynamics over time. This approach enabled recruitment of a large and diverse convenience sample, facilitating a thorough investigation of HPV-related health outcomes among study participants. The data collected were analysed using rigorous statistical methods to estimate the burden of oral HPV among Aboriginal and Torres Strait Islander (ATSI) populations [18]. Details of the data collection process are presented in the participant flow chart in Supplementary Figure S1 [18].

### 2.4. DNA Analysis

Saliva specimens were obtained at baseline and at the 12- and 24-month follow-up visits using OMNIgene^®^ Discover kits (OM-501; DNA Genotek Inc., Ottawa, ON, Canada). A total of 473 participants had oral HPV measurements available at all three time points. DNA was subsequently isolated using the Promega Maxwell^®^ Viral Kit. To assess sample adequacy and DNA quality, β-globin PCR with PCO3/PCO4 primers was performed for every specimen. This step confirmed the presence of human DNA and helped identify potential PCR inhibition. Samples that tested negative for β-globin were considered unsuitable and were excluded from subsequent analyses. HPV DNA was identified using a nested PCR approach incorporating MY09/11 and GP5+/6+ primers, which enables detection of a wide range of mucosal HPV types, including oncogenic high-risk types and HPV types associated with Heck’s disease. Samples positive for HPV DNA underwent sequencing for confirmation and genotype identification. PCR products were purified using the Agencourt^®^ AMPure PCR Purification Kit with a magnetic 96-ring SPRIplate^®^, followed by sequencing reactions containing purified products, GP+ primer, and BigDye Terminator. Sequencing products were further cleaned using the Agencourt^®^ CleanSEQ dye-terminator removal kit. Direct sequencing was performed initially; specimens containing multiple HPV types were cloned, with at least five clones sequenced per sample. Sequences were analysed using an ABI 3100 automated DNA sequencer and matched against reference sequences in GenBank using NCBI BLASTn.(BLAST+ version 2.10.1; National Center for Biotechnology Information, Bethesda, MD, USA). This PCR-based approach has been applied previously and demonstrated good reliability and reproducibility [19,20].

A total of 45 HPV types were identified: 3, 6, 7, 10, 11, 13, 16, 18, 26, 30, 31, 32, 33, 34, 35, 39, 40, 42, 44, 45, 51, 52, 53, 54, 56, 58, 59, 62, 66, 67, 68, 69, 72, 73, 76, 81, 82, 84, 87, 89, 90, 103, 106, 107, and 182. For the final analysis, all detected HPV types were combined and analysed as “any HPV.” HPV types were not analysed separately according to low-risk HPV, high-risk HPV, or HPV-13 and HPV-32 categories [18].

### 2.5. Variables

Covariates were selected from the self-reported questionnaire and included sociodemographic characteristics (age, gender, geographic location, highest educational attainment, income, ownership of a government-administered health care card [HCC], and car ownership); self-rated general and oral health; health-related behaviours (smoking status and recreational drug use); self-reported HPV vaccination status; and sexual behaviours were assessed using two questions: (1) “Have you ever given oral sex?” and (2) “Have you ever received oral sex?” Responses were dichotomised as “Yes” if participants reported ever giving and/or receiving oral sex and “No” if they reported neither. Age at first giving and/or receiving oral sex was also recorded.

The individual participant was the unit of analysis, and longitudinal HPV outcomes were assessed at baseline, 12 months and 24 months. Incident infection was defined as any HPV type detection at follow-up among participants who were HPV-negative at baseline and/or at 12 months. Persistence was defined as the detection of any HPV type at consecutive assessment points. Clearance included participants who were oral HPV positive at baseline and/or 12 months and subsequently became HPV negative at the following assessment. This category also included fluctuating patterns of HPV detection, specifically: (1) HPV positive at baseline, negative at 12 months, and positive again at 24 months; or (2) HPV negative at baseline, positive at 12 months, and negative again at 24 months.

### 2.6. Statistical Analysis

Analyses began with descriptive statistics summarising the number and prevalence of incident, persistence, and cleared oral HPV detection, stratified by residential location. Multivariable log-binomial regression models were then used to identify factors independently associated with the primary outcomes, incidence and persistence of any oral HPV detection, and to estimate crude and adjusted risk ratios (RRs) with 95% confidence intervals (CIs). Covariates were selected a priori based on conceptual relevance and evidence from previous literature regarding factors potentially associated with HPV acquisition and persistence, rather than solely on statistical significance in univariable analyses. Five models were fitted: Model 1, the crude model; Model 2, adjusted for sociodemographic factors; Model 3, adjusted for health and health-related behaviours; Model 4, adjusted for sexual behaviours; and Model 5, the fully adjusted model incorporating covariates from the preceding models. For analyses involving small sample sizes, modified Poisson regression with robust standard errors was used to estimate risk ratios (RRs) and 95% confidence intervals.

Data files were managed, summary variables were computed, and statistical analyses were conducted using SAS software version 9.4 (SAS Institute Inc., Cary, NC, USA).

## 3. Results

Table 1a,b show the baseline to 12- and 24-month sample characteristics and association with incidence, persistence and clearance of any oral HPV detection among non-metropolitan (n = 482) and metropolitan (n = 298) Indigenous Australian adults. Close to half of the participants were aged 37 years or older. Only 30% of participants were male; 80% of respondents had a Health Care Card, and only 30% had achieved higher than high school education. Over 80% had self-reported their general health to be good to excellent, and just under 70% had self-reported their oral health to be good to excellent. Only 8.4% of respondents were aware they had received at least one HPV vaccination.

### 3.1. Baseline Characteristics (Non-Metropolitan)

Among participants living in non-metropolitan areas, incident oral HPV detection was lower among those who self-reported a previous HPV infection (7.7%) than among those who reported no previous HPV infection (26.6%) or were unsure of their previous HPV status (23.9%). However, this finding was based on only one participant reporting a previous HPV infection and should therefore be interpreted with considerable caution. A lower incidence of oral HPV detection was also observed among participants who reported first giving oral sex before 16 years of age (14.0%) compared with those who first gave oral sex at age 16 years or older (31.8%) (Table 1a). Given the small subgroup sizes and the exploratory nature of these analyses, this association should also be interpreted cautiously.

### 3.2. Baseline Characteristics (Metropolitan)

Among metropolitan Indigenous adults (Table 1b), 157 (55.9%) were current smokers, 42 (14.9%) were ex-smokers, and 82 (29.2%) had never smoked; smoking status was missing for 14 participants. Incident oral HPV detection was observed in 31.0% of ex-smokers, compared with 24.8% of current smokers and 15.9% of never-smokers. Clearance was also highest among ex-smokers, with 20 of 42 participants (47.6%) experiencing clearance, compared with 26.8% of current smokers and 25.6% of never-smokers. However, estimates for ex-smokers were based on a relatively small subgroup and should therefore be interpreted cautiously.

Among participants who responded to the question on age at first giving oral sex, 23.0% reported first giving oral sex before 16 years of age and 77.0% at age 16 years or older; responses were missing for 98 participants. No statistically significant differences in incident or cleared oral HPV detection were observed between these age groups. Persistent oral HPV detection was observed in 23.9% of participants who first gave oral sex before age 16, compared with 9.1% of those who first gave oral sex at age 16 years or older (Table 1b).

Table 2a,b are summaries of the multivariable association between incidence of Oral HPV detection and risk factors among non-metropolitan and metropolitan Indigenous Australian adults. The five models previously described were used for analysis. Associations were presented as risk ratios along with their 95% confidence intervals, and bold were identified as statistically significant differences.

For non-metropolitan Indigenous Australian adults (Table 2a), there was a significantly higher risk of incidence of HPV infection for those who were ex-users of recreational drugs (Model 5: RR = 1.74, 95% CI: 1.01–3.62), although this was not supported by other models. Compared with participants who reported giving oral sex at age 16 or older, those who reported giving oral sex before age 16 had a lower risk of incident HPV infection across Models 1, 4, and 5, risk ratio was 0.44, 0.33 and 0.30, respectively.

For Metropolitan residing Indigenous Australian adults (Table 2b), there was a significantly higher risk of HPV infection incidence for those who were ex-smokers (Model 1: 1.95, Model 3: 1.95, Model 5: RR = 2.90, 95% CI: 1.02–8.60), and current smokers (Model 5: 2.65), than those who had never smoked. There was a reduced rate of incidence of HPV for those who were unsure of their HPV vaccination status (Model 5: RR = 0.3, 95% CI: 0.10–0.95).

Table 3a,b are multivariable log-binomial regression models association between persistence of Oral HPV detection and risk factors among non-metropolitan and metropolitan Indigenous Australian adults. The five models previously described were used for analysis. Associations were presented as risk ratios along with their 95% confidence intervals, and bold were identified as statistically significant differences.

For those living in Non-Metropolitan locations (Table 3a), older age was significantly associated with a higher risk of persistent Oral HPV detection (Model 5: RR = 2.79, 95% CI: 1.11–7.00), indicating age as a crucial factor in infection persistence. Ex-users of recreational drugs had a significantly lower risk ratio compared to never users (Model 3: 0.56). This suggests that cessation of drug use may reduce the risk of persistent Oral HPV detections.

For those residing in metropolitan locations (Table 3b), there was a significantly higher association of persistent HPV infections in Indigenous Australian adults who had first given oral sex before the age of 16 (Model 1: 2.63, Model 4: 2.72, Model 5: RR = 2.82, 95% CI: 1.08–11.73).

There was also a significantly higher association for persistent HPV infections for metropolitan Indigenous Australian adults who had ever received oral sex (Model 1: 3.15).

Early initiation of giving oral sex is associated with a significantly higher prevalence ratio for persistent Oral HPV detections. This suggests that early exposure to sexual activities might increase the risk of persistent infections.

Receiving oral sex is significantly associated with a higher risk of persistent Oral HPV detection, indicating the role of sexual practices in infection persistence [20].

## 4. Discussion

Although several behavioural factors were associated with oral HPV detection in both residential groups, the pattern of associations differed between metropolitan and non-metropolitan participants. Smoking was associated with higher incidence and persistence of oral HPV detection in some analyses in both groups. Among non-metropolitan participants, older age (≥37 years) was associated with persistent oral HPV detection, while former recreational drug use was associated with a lower risk of persistence. Earlier initiation of giving oral sex (<16 years) was associated with lower incidence but higher persistence in this group. In contrast, among metropolitan participants, receiving oral sex was associated with an increased risk of persistent oral HPV detection, while the associations with age and former recreational drug use observed in the non-metropolitan group were not evident. These differences suggest that the factors associated with oral HPV detection may vary by residential setting; however, residential location itself should not be interpreted as a causal factor. Given the observational design, small subgroup sizes, potential residual confounding, and multiple comparisons, these findings should be regarded as exploratory and interpreted cautiously.

Uncertainty about HPV vaccination status was associated with a lower incidence of oral HPV detection in the metropolitan population. However, this finding should be interpreted cautiously and may reflect recall bias, exposure misclassification, residual confounding, or chance findings rather than any protective effect.

The analysis highlights the importance of smoking status, age, recreational drug use, sexual behaviours, and HPV vaccination in understanding the risk factors associated with the incidence and persistence of oral HPV infections among Indigenous Australian adults. These findings may help inform future research and public health planning; however, the observational nature of the study prevents conclusions regarding the effectiveness of specific interventions.

Both current and former smoking were associated with higher risks of incident and persistent oral HPV detection in some analyses, consistent with previous studies reporting an association between smoking and persistent HPV detection [21,22]. Several biological mechanisms have been proposed to explain this relationship. Tobacco smoke may impair local and systemic immune responses, potentially reducing the ability to clear HPV [23,24,25]. In addition, nicotine and other tobacco-related compounds may alter the local immune environment and induce cellular and molecular changes in oral epithelial tissues, which could facilitate HPV persistence [23,26]. Tobacco-related exposure has also been linked to DNA damage and disruption of cellular pathways involved in carcinogenesis [26]. However, these biological mechanisms were not directly assessed in the present study. Therefore, the observed associations between smoking and oral HPV detection should be interpreted cautiously and should not be considered evidence of a causal relationship.

Individuals aged 37 years and older living in non-metropolitan locations had a higher risk of persistent oral HPV infection. Several factors may contribute to this association, including cumulative HPV exposure, reactivation of previously acquired or latent infections, smoking and other health-related behaviours, differences in access to care, cohort effects, and age-related changes in immune function [27,28,29,30]. Previous studies have suggested that declining immune function with age may reduce the ability to clear HPV infections and may facilitate reactivation of latent infection; however, immune function was not directly assessed in the present study, and these potential mechanisms therefore remain speculative [31]. Further research is needed to better understand age-related differences in oral HPV detection and persistence among Aboriginal and Torres Strait Islander populations and to inform appropriately targeted prevention strategies.

Age at first oral sex was associated with different patterns of oral HPV detection across residential groups. Among metropolitan participants, those who first gave oral sex before 16 years of age had a higher prevalence of persistent oral HPV detection than those who first gave oral sex at age 16 years or older (23.9% vs. 9.1%). In contrast, among non-metropolitan participants, those who first gave oral sex before age 16 had a lower incidence of oral HPV detection than those who first gave oral sex at age 16 years or older (14.0% vs. 31.8%). The reason for these differing patterns is unclear and may reflect differences in sexual behaviours, partner characteristics, or other unmeasured social and behavioural factors. Given the relatively small subgroup sizes, potential residual confounding, and multiple comparisons, these findings should be considered exploratory and interpreted cautiously [32].

Early exposure to HPV during adolescence, when the immune system is still developing, may result in a higher likelihood of persistent infection due to an immature immune response [33,34]. Furthermore, early sexual initiation is often associated with a higher number of sexual partners over a lifetime, increasing the risk of HPV exposure and persistence [35].

Early sexual activity can expose individuals to HPV when their immune systems are not fully developed, heightening the likelihood of persistent infections. Studies have shown that early sexual debut is linked to a higher number of sexual partners, which elevates the risk of HPV infection and its persistence. Comprehensive sexual education programs that address the risks associated with early sexual activity and promote safer sexual practices are crucial in reducing the incidence and persistence of HPV [10,36].

Recreational drug use also shows differing impacts between the two populations. In non-metropolitan areas, ex-users of recreational drugs have a significantly lower risk of persistent HPV infections compared to those who have never taken recreational drugs. This finding should be interpreted cautiously. The study did not collect information regarding drug type, duration or intensity of use, time since cessation, or immune status, and therefore the mechanisms underlying this association cannot be determined.

Recreational drug use is often linked to behaviours associated with HPV exposure, including higher numbers of sexual partners and inconsistent condom use. Changes in health behaviours following cessation of recreational drug use may warrant further investigation; however, the present study did not collect sufficient information to examine these pathways directly. Further research is required to determine whether the observed association reflects behavioural differences, residual confounding, chance findings, or other unmeasured factors.

A systematic review reported a 68% reduction in high-risk HPV (hrHPV) infection in countries where vaccination coverage reached at least 50% [37]. Improving awareness and knowledge of the benefits, safety, and effectiveness of HPV vaccination may increase vaccine uptake and coverage and ultimately reduce HPV-related infections and cancers. In the present study, similar proportions of participants in metropolitan and non-metropolitan areas reported low vaccination uptake. However, self-reported vaccination status may be inaccurate [38]. Therefore, the lower incidence of oral HPV detection observed among participants who were uncertain of their vaccination status should not be interpreted as evidence of a protective effect and may instead reflect recall bias, misclassification, residual confounding, or small numbers of events. Culturally appropriate and accessible vaccination and education programs are particularly important for Indigenous communities, as they may help address misinformation, improve knowledge, reduce logistical barriers, and support greater vaccine uptake. Previous studies have shown that targeted educational approaches can improve vaccination coverage and contribute to better community health outcomes [39].

Socioeconomic variables did not demonstrate strong or consistent independent associations with oral HPV detection outcomes in the present analyses. Although broader social determinants remain important contributors to health inequities experienced by Aboriginal and Torres Strait Islander peoples, conclusions regarding socioeconomic influences on oral HPV detection should be interpreted cautiously based on the findings of this study.

Strengths of this study include its longitudinal cohort design with follow-up at 12 and 24 months, enabling assessment of changes in oral HPV detection over time, including incidence, persistence, and clearance. Recruitment of a relatively large sample of Aboriginal and Torres Strait Islander adults through Aboriginal Community Controlled Health Organisations, with involvement of Indigenous research staff, enhanced the cultural appropriateness and relevance of the study.

Several limitations should be considered. Convenience sampling may have introduced selection bias and limits generalisability, while the higher proportion of women may reduce applicability to men, who experience a greater burden of HPV-related oropharyngeal cancer in many populations [5,10]. Self-reported smoking, recreational drug use, sexual behaviours, previous HPV infection, and vaccination status may be affected by recall or social desirability bias, and vaccination status was not independently verified. Several subgroup analyses, including those involving ex-smokers, were based on small numbers of participants and outcome events, resulting in potentially unstable estimates, limited precision, and wide confidence intervals. Some associations, including those involving age at first oral sex, arose from secondary analyses and may represent chance findings given the number of comparisons performed. Detection of HPV DNA in saliva does not necessarily indicate infection at a specific oral anatomical site. Persistence defined using any HPV type may not reflect persistence of the same genotype, while inclusion of fluctuating detection patterns in the clearance definition may have led to misclassification or underestimation of incidence and persistence. Residual confounding remains possible, and attrition across follow-up may also have introduced differential loss-to-follow-up bias.

## 5. Conclusions

This study provides longitudinal evidence on saliva-based oral HPV detection among Aboriginal and Torres Strait Islander adults in South Australia. Differences in the observed patterns of incident, persistent, and cleared oral HPV detection were identified between metropolitan and non-metropolitan participants, and several sociodemographic and behavioural factors were associated with these outcomes. These findings should not be interpreted as evidence that residential location or the identified behavioural factors caused the observed differences. Given the observational design, convenience sampling, small numbers in some subgroups, and potential for residual confounding, the results should be considered hypothesis-generating. They may help identify factors that warrant further investigation but do not establish the effectiveness of specific public health interventions. Further longitudinal studies with larger samples and genotype-specific HPV assessment are needed to better understand oral HPV detection patterns, persistence, and associated factors among Aboriginal and Torres Strait Islander populations.

## Figures and Tables

**Table 1:** (**a**) Baseline sample characteristics and association with incidence, persistence and clearance of any Oral HPV detection among non-metropolitan Indigenous Australian adults (n = 482). (**b**) Baseline sample characteristics and association with incidence, persistence, and clearance of any Oral HPV detection among metropolitan Indigenous Australian adults (n = 298).

(**a**)								
	**Overall**		**Incidence**		**Persistent**		**Clearance**	
	**N**	**% (95% CI)**	**N**	**% (95% CI)**	**N**	**% (95% CI)**	**N**	**% (95% CI)**
Total	482		123	25.5 (21.6–29.4)	88	18.3 (14.8–21.7)	128	26.6 (22.6–30.5)
Age group (years)								
≥37	263	54.6 (50.1–59.0)	63	24.0 (18.8–29.1)	52	19.8 (14.9–24.6)	74	28.1 (22.7–33.6)
<37	219	45.4 (41.0–49.9)	60	27.4 (21.5–33.3)	36	16.4 (11.5–21.4)	54	24.7 (18.9–30.4)
Missing	0		0		0		0	
Sex								
Male	147	**30.5 (26.4–34.6)**	36	24.5 (17.5–31.5)	21	14.3 (8.6–20.0)	44	29.9 (22.5–37.4)
Female	335	**69.5 (65.4–73.6)**	87	26.0 (21.3–30.7)	67	20.0 (15.7–24.3)	84	25.1 (20.4–29.7)
Missing	0		0		0		0	
Education level								
High school or less	334	**70.0 (65.9–74.1)**	90	26.9 (22.2–31.7)	63	18.9 (14.7–23.1)	90	26.9 (22.2–31.7)
Trade or over	143	**30.0 (25.9–34.1)**	33	23.1 (16.1–30.0)	25	17.5 (11.2–23.7)	38	26.6 (19.3–33.8)
Missing	5		0		0		0	
Income								
Centrelink	361	**75.8 (72.0–79.7)**	94	26.9 (21.5–30.6)	69	19.1 (15.0–23.2)	93	25.6 (21.2–30.3)
Job	115	**24.2 (20.3–28.0)**	28	24.3 (16.5–32.2)	19	16.5 (9.7–23.3)	33	28.7 (20.4–37.0)
Missing	6		1		0		2	
Health care card								
Yes	367	**80.0 (76.3–83.6)**	96	26.2 (21.6–30.7)	69	18.8 (14.8–22.8)	96	26.2 (21.6–30.7)
No	92	**20.0 (16.4–23.7)**	23	25.0 (16.1–33.9)	17	18.5 (10.5–26.4)	28	30.4 (21.0–39.9)
Missing	23		4		2		4	
Car ownership								
No	215	**44.9 (40.4–49.4)**	61	28.4 (22.3–34.4)	41	19.1 (13.8–24.3)	62	28.8 (22.8–34.9)
Yes	264	**55.1 (50.6–59.6)**	62	23.5 (18.4–28.6)	46	17.4 (12.8–22.0)	66	25.0 (19.8–30.2)
Missing	3		0		1		0	
Smoke status								
Current smoker	278	**60.3 (55.8–64.8)**	63	22.7 (17.7–27.6)	52	18.7 (14.1–23.3)	67	24.1 (19.1–29.1)
Ex-smoker	54	**11.7 (8.8–14.7)**	17	31.5 (19.0–43.9)	8	14.8 (5.3–24.3)	14	25.9 (14.2–37.7)
Never smoked	129	**28.0 (23.9–32.1)**	36	27.9 (20.1–35.7)	25	19.4 (12.5–26.2)	36	27.9 (20.1–35.7)
Missing	21		7		3		11	
Recreational drug use								
Current user	96	**20.3 (16.7–23.9)**	23	24.0 (15.4–32.5)	19	19.8 (11.8–27.8)	26	27.1 (18.2–36.0)
Ex-user	153	**32.3 (28.1–36.6)**	42	27.5 (20.4–34.5)	20	13.1 (7.7–18.4)	36	23.5 (16.8–30.3)
Never used	224	**47.4 (42.8–51.9)**	57	25.4 (19.7–31.2)	48	21.4 (16.0–26.8)	64	28.6 (22.6–34.5)
Missing	9		1		1		2	
Self-rated general health								
Fair/Poor	86	**18.1 (14.7–21.6)**	18	20.9 (12.3–30.0)	11	12.8 (5.7–19.9)	23	26.7 (17.4–36.1)
Excellent/very good/Good	388	**81.9 (78.4–85.3)**	104	26.8 (22.4–31.2)	76	19.6 (15.6–23.6)	103	26.5 (22.1–31.0)
Missing	8		1		1		2	
Self-rated oral health								
Fair/Poor	155	**33.1 (28.8–37.4)**	44	28.4 (21.3–35.5)	26	16.8 (10.9–22.7)	40	25.8 (18.9–32.7)
Excel/very good/Good	313	**66.9 (62.6–71.2)**	78	24.9 (20.1–29.7)	60	19.2 (14.8–23.5)	84	26.8 (21.9–31.8)
Missing	14		1		2		4	
Had HPV infection								
Yes	13	**2.7 (1.3–4.2)**	1	**7.7 (0.0–22.1)**	3	23.1 (0.1–46.1)	1	7.7 (0.0–22.2)
No	372	**78.0 (74.3–81.7)**	99	**26.6 (22.2–31.1)**	63	16.9 (13.1–20.8)	101	27.2 (22.6–31.7)
Don’t know	92	**19.3 (15.7–22.8)**	22	23.9 (15.2–32.7)	21	22.8 (14.2–31.4)	25	27.2 (18.1–36.3)
Missing	5		1		1		1	
Ever received HPV vaccination								
Yes	40	**8.4 (5.9–10.8)**	11	27.5 (13.6–41.4)	4	10.0 (0.7–19.3)	7	17.5 (5.7–29.3)
No	273	**57.0 (52.5–61.4)**	71	26.0 (20.8–31.2)	56	20.5 (15.7–25.3)	79	28.9 (23.5–34.3)
Don’t know	166	**34.7 (30.4–38.9)**	41	24.7 (18.1–31.3)	27	16.3 (10.6–21.9)	42	25.3 (18.7–31.9)
Missing	3		0		1		0	
Even given oral sex								
Yes	263	**61.2 (56.5–65.8)**	73	27.8 (22.3–33.2)	44	16.7 (12.2–21.3)	70	26.6 (21.3–32.0)
No	167	**38.8 (34.2–43.5)**	43	25.7 (19.0–32.4)	30	18.0 (12.1–23.8)	41	24.6 (18.0–31.1)
Missing	52		7		14		17	
Age at first giving oral sex (Years)								
<16	57	**22.1 (17.0–27.2)**	8	**14.0 (5.0–23.1)**	11	19.3 (9.0–29.6)	14	24.6 (13.3–35.8)
≥16	201	**77.9 (72.8–83.0)**	64	**31.8 (25.4–38.3)**	33	16.4 (11.3–21.6)	53	26.4 (20.2–32.5)
Missing	224		51		44		61	
No. of oral sex given								
>3	100	**38.5 (32.5–44.4)**	29	29.0 (20.0–38.0)	16	16.0 (8.8–23.2)	27	27.0 (18.2–35.8)
≤3	160	**61.5 (55.6–67.5)**	42	26.3 (19.4–33.1)	28	17.5 (11.6–23.4)	42	26.3 (19.4–33.1)
Missing	222		52		44		59	
Ever received oral sex								
Yes	257	**60.5 (55.8–65.1)**	69	26.8 (21.4–32.3)	37	14.4 (10.1–18.7)	69	26.8 (21.4–32.3)
No	168	**39.5 (34.9–44.2)**	44	26.2 (19.5–32.9)	35	20.8 (14.7–27.0)	41	24.4 (17.9–30.9)
Missing	57		10		16		18	
Age at first received oral sex (Years)								
<16	59	**23.3 (18.1–28.6)**	11	18.6 (8.6–28.6)	6	10.2 (2.4–17.9)	17	28.8 (17.2–40.4)
≥16	194	**76.7 (71.4–81.9)**	59	29.9 (23.4–36.4)	31	16.0 (10.8–21.2)	50	25.8 (19.6–32.0)
Missing	229		54		51		67	
No. of times oral sex received								
>3	118	**46.3 (40.1–52.4)**	32	27.1 (19.0–35.2)	19	16.0 (9.4–22.8)	32	27.1 (19.0–35.2)
≤3	137	**53.7 (47.6–59.9)**	37	27.0 (19.5–34.5)	18	13.1 (7.4–18.8)	35	25.5 (18.2–32.9)
Missing	227		54		51		61	
Current relationship status								
Single	231	**53.1 (48.4–57.8)**	45	25.0 (18.6–31.4)	33	18.3 (12.7–24.0)	45	25.0 (18.6–31.4)
Short-term	24	**5.5 (3.3–7.7)**	8	33.3 (14.4–52.3)	4	16.7 (1.7–31.6)	7	29.2 (10.9–47.4)
Stable and long	180	**41.4 (36.7–46.0)**	62	26.8 (21.1–32.6)	39	16.9 (12.0–21.7)	61	26.4 (20.7–32.1)
Missing	47		8		12		15	
(**b**)								
	**Overall**		**Incidence**		**Persistent**		**Clearance**	
	**N**	**% (95% CI)**	**N**	**% (95% CI)**	**N**	**% (95% CI)**	**N**	**% (95% CI)**
Total	298		69	23.2 (18.3–28.0)	35	11.7 (8.1–15.4)	87	29.2 (24.0–34.4)
Age group (years)								
≥37	152	51.0 (45.3–56.7)	36	23.7 (16.9–30.5)	18	11.8 (6.7–17.0)	43	28.3 (21.1–35.5)
<37	146	49.0 (43.3–54.7)	33	22.6 (15.8–29.4)	17	11.6 (6.4–16.9)	44	30.1 (22.7–37.6)
Missing	0		0		0		0	
Sex								
Male	98	**32.9 (27.5–38.3)**	28	28.6 (19.6–37.6)	13	13.3 (6.5–20.0)	24	24.5 (15.9–33.1)
Female	200	**67.1 (61.7–72.5)**	41	20.5 (14.9–26.1)	22	11.0 (6.6–15.4)	63	31.5 (25.0–38.0)
Missing	0		0		0		0	
Education level								
High school or less	174	**59.8 (54.1–65.5)**	39	22.4 (16.2–28.6)	25	14.4 (9.1–19.6)	50	28.7 (22.0–35.5)
Trade or over	117	**40.2 (34.5–45.9)**	29	24.8 (16.9–32.7)	10	8.5 (3.5–13.6)	37	31.6 (23.1–40.1)
Missing	7		1		0		0	
Income								
Centrelink	208	**71.5 (66.3–76.7)**	46	22.1 (16.4–27.7)	27	13.0 (8.4–17.6)	66	31.7 (25.4–38.1)
Job	83	**28.5 (23.3–33.7)**	21	25.3 (15.9–34.7)	8	9.6 (3.3–16.0)	20	24.1 (14.8–33.4)
Missing	7		2		0		2	
Health care card								
Yes	211	**74.8 (69.7–79.9)**	43	20.4 (14.9–25.8)	28	13.3 (8.7–17.9)	62	29.4 (23.2–35.6)
No	71	**25.2 (20.1–30.3)**	20	28.2 (17.6–38.7)	6	8.5 (1.9–15.0)	20	28.2 (17.6–38.7)
Missing	16		5		1		5	
Car ownership								
No	108	**36.6 (31.1–42.1)**	28	25.9 (17.6–34.2)	15	13.9 (7.3–20.4)	39	36.1 (27.0–45.2)
Yes	187	**63.4 (57.9–68.9)**	40	21.4 (15.5–27.3)	20	10.7 (6.2–15.2)	48	25.7 (19.4–32.0)
Missing	3		1		0		0	
Smoking status								
Current smoker	157	**55.9 (50.0–61.7)**	39	24.8 (18.0–31.6)	18	11.5 (6.5–16.5)	42	**26.8 (19.8–33.4)**
Ex-smoker	42	**14.9 (10.8–19.1)**	13	**31.0 (20.9–45.0)**	4	9.5 (0.6–18.5)	20	**47.6 (33.7–62.8)**
Never smoked	82	**29.2 (23.8–34.5)**	13	**15.9 (7.8–20.8)**	11	13.4 (6.0–20.8)	21	25.6 (16.1–35.1)
Missing	17		4		2		4	
Recreational drug use								
Current user	60	**20.3 (15.7–25.0)**	10	16.7 (7.2–26.2)	10	16.7 (7.2–26.2)	21	35.0 (22.9–47.1)
Ex-user	102	**34.6 (29.1–40.0)**	27	26.5 (17.9–35.1)	12	11.8 (5.5–18.1)	31	30.4 (21.4–39.4)
Never used	133	**45.1 (39.4–50.8)**	31	23.3 (16.1–30.5)	13	9.8 (4.7–14.9)	34	25.6 (18.1–33.0)
Missing	3		1		0		1	
Self-rated general health								
Fair/Poor	86	**29.9 (24.5–35.2)**	19	22.1 (13.3–30.9)	10	11.6 (4.8–18.4)	19	22.1 (13.3–30.9)
Excellent/very good/Good	202	**70.1 (64.8–75.5)**	47	23.3 (17.4–29.1)	24	11.9 (7.4–16.4)	67	33.2 (26.6–39.7)
Missing	10		3		1		1	
Self-rated oral health								
Fair/Poor	105	**36.6 (31.0–42.2)**	25	23.8 (15.6–32.0)	14	13.3 (6.8–19.9)	28	26.7 (18.2–35.2)
Excellent/very good/Good	182	**63.4 (57.8–69.0)**	40	22.0 (15.9–28.0)	20	11.0 (6.4–15.6)	57	31.3 (24.5–38.1)
Missing	11		4		1		2	
Had HPV infection								
Yes	6	**2.0 (0.4–3.7)**	2	33.3 (0.0–71.3)	1	16.7 (0.0–46.7)	1	16.7 (0.0–46.7)
No	247	**84.0 (79.8–88.2)**	57	23.1 (17.8–28.4)	31	12.6 (8.4–16.7)	72	29.1 (23.4–34.9)
Don’t know	41	**36.2 (30.6–41.7)**	8	19.5 (7.3–31.7)	2	4.9 (0.0–11.5)	13	31.7 (17.4–46.0)
Missing			2		1		1	
Ever received HPV vaccination								
Yes	23	**7.8 (4.8–10.9)**	8	34.8 (15.2–54.4)	3	13.0 (0.0–26.9)	9	39.1 (19.1–59.2)
No	164	**56.0 (50.3–61.7)**	40	24.4 (17.8–31.0)	24	14.6 (9.2–20.1)	46	28.0 (21.1–35.0)
Don’t know	106	**36.2 (30.6–41.7)**	19	17.9 (10.6–25.3)	7	6.6 (1.8–11.4)	31	29.2 (20.5–38.0)
Missing	5		2		1		1	
Ever given oral sex								
Yes	202	**77.1 (72.0–82.2)**	42	20.8 (15.2–26.4)	25	12.4 (7.8–16.9)	59	29.2 (22.9–35.5)
No	60	**22.9 (17.8–28.0)**	16	26.7 (15.4–37.9)	5	8.3 (1.3–15.4)	19	31.7 (19.8–43.5)
Missing	36		11		5		9	
Age first given oral sex (Years)								
<16	46	**23.0 (17.1–28.9)**	10	21.7 (9.7–33.8)	11	**23.9 (13.7–36.3)**	10	21.7 (9.7–33.8)
≥16	154	**77.0 (71.1–82.9)**	31	20.1 (13.7–26.5)	14	**9.1 (4.5–13.5)**	48	31.2 (23.8–38.5)
Missing	98		28		10		29	
No. of times oral sex given								
>3	101	50.5 (43.5–57.5)	21	20.8 (12.8–28.8)	15	14.9 (7.9–21.8)	28	27.7 (18.9–36.5)
≤3	99	49.5 (42.5–56.5)	21	21.2 (13.1–29.3)	10	10.0 (4.1–16.1)	30	30.3 (21.2–39.4)
Missing	98		27		10		29	
Ever received oral sex								
Yes	206	**80.5 (75.6–85.4)**	43	20.9 (15.3–26.5)	26	**12.6 (9.5–17.2)**	60	29.1 (22.9–35.4)
No	50	**19.5 (14.6–24.4)**	13	26.0 (13.8–38.2)	2	**4.0 (0.0–9.1)**	16	32.0 (19.0–45.0)
Missing	42		13		7		11	
Age first received oral sex (Years)								
<16	64	**31.1 (24.7–37.4)**	16	25.0 (14.3–35.7)	12	18.8 (9.1–28.4)	22	34.4 (22.6–46.1)
≥ 16	142	**68.9 (62.6–75.3)**	27	19.0 (12.5–25.5)	14	9.9 (4.9–14.8)	38	26.7 (19.4–34.1)
Missing	92		26		9		27	
No. of times oral sex received								
>3	104	**51.0 (44.1–57.9)**	24	23.1 (14.9–31.2)	16	15.4 (8.4–22.4)	32	30.8 (21.8–39.7)
≤3	100	**49.0 (42.1–55.9)**	19	19.0 (11.2–26.8)	10	10.0 (4.1–15.9)	28	28.0 (19.1–36.9)
Missing	94		26		9		27	
Current relationship status								
Single	115	**44.2 (38.2–50.3)**	25	21.7 (14.2–29.3)	15	13.0 (6.8–19.2)	32	27.8 (19.6–36.1)
Short-term	10	**3.8 (1.5–6.2)**	2	20.0 (0.0–45.0)	2	20.0 (0.0–45.0)	3	30.0 (1.4–58.6)
Stable and long	135	**51.9 (45.8–58.0)**	31	23.0 (15.8–30.1)	14	10.4 (5.2–15.5)	42	31.1 (23.3–39.0)
Missing	38		11		4		10	

Notes: (**a**) Bold values indicate statistically significant associations. (**b**) Bold were donated as statistically significant difference.

**Table 2:** (**a**) Multivariable association between incidence of Oral HPV detection and risk factors among non-metropolitan Indigenous Australian adults (n = 482). (**b**) Multivariable association between incidence of Oral HPV detection and risk factors among metropolitan Indigenous Australian adults (n = 298).

(**a**)					
	**Model 1**	**Model 2**	**Model 3**	**Model 4**	**Model 5**
	**Risk Ratio (95% CI)**				
Age group (years)					
≥37	0.87 (0.61–1.25)	0.87 (0.61–1.26)			0.94 (0.51–1.73)
<37	ref	ref			ref
Sex					
Male	0.94 (0.64–1.39)	0.96 (0.65–1.43)			1.02 (0.52–2.01)
Female	ref	ref			ref
Education level					
High school or less	1.17 (0.78–1.74)	1.20 (0.79–1.83)			1.29 (0.72–2.30)
Trade or over	ref	ref			ref
Income					
Centrelink	1.07 (0.70–1.63)	0.96 (0.54–1.69)			1.54 (0.73–2.30)
Job	ref	ref			ref
Health care card					
Yes	1.05 (0.66–1.65)	1.01 (0.57–1.79)			0.59 (0.26–1.35)
No	ref	ref			ref
Car ownership					
No	1.21 (0.85–1.72)	1.14 (0.78–1.68)			1.42 (0.78–2.58)
Yes	ref	ref			ref
Smoking status					
Current smoker	0.81 (0.54–1.22)		0.83 (0.53–1.29)		0.70 (0.36–1.39)
Ex-smoker	1.13 (0.63–2.00)		1.05 (0.56–1.94)		0.60 (0.23–1.58)
Never smoked	ref		ref		ref
Recreational drug use					
Current user	0.94 (0.58–1.53)		0.98 (0.57–1.69)		0.90 (0.37–2.21)
Ex-user	1.08 (0.72–1.61)		1.18 (0.76–1.82)		**1.74 (1.01–3.62)**
Never used	ref		ref		ref
Self-rated general health					
Fair/Poor	0.78 (0.47–1.29)		0.73 (0.41–1.28)		0.83 (0.38–1.78)
Excellent/very good/Good	ref		ref		ref
Self-rated oral health					
Fair/Poor	1.14 (0.79–1.65)		1.19 (0.78–1.81)		0.98 (0.53–1.83)
Excellent/very good/Good	ref		ref		ref
Had HPV infection					
Yes	0.29 (0.04–2.07)		0.32 (0.04–2.35)		0.29 (0.03–2.35)
Don’t know	0.90 (0.57–1.43)		0.92 (0.54–1.56)		ref
No	ref		ref		
Ever received HPV vaccination					
No	0.95 (0.50–1.78)		0.89 (0.46–1.69)		1.15 (0.52–2.57)
Don’t know	0.90 (0.46–1.74)		0.86 (0.43–1.72)		1.05 (0.42–2.66)
Yes	ref		ref		ref
Ever given oral sex					
Yes	1.08 (0.74–1.57)			-	-
No	ref			-	-
Age first given oral sex (Years)					
<16	**0.44 (0.21–0.92)**			**0.33 (0.12–0.93)**	**0.30 (0.09–0.93)**
≥16	ref			ref	ref
No. of times oral sex given					
>3	1.10 (0.69–1.77)			1.14 (0.66–3.03)	1.61 (0.61–4.29)
≤3	ref			ref	ref
Ever received oral sex					
Yes	1.03 (0.70–1.50)			-	-
No	ref			-	-
Age first received oral sex (Years)					
<16	0.62 (0.33–1.19)			1.08 (0.46–2.50)	1.08 (0.42–2.81)
≥16	ref			ref	ref
No. of times oral sex received					
>3	1.00 (0.63–1.61)			0.78 (0.36–1.68)	0.65 (0.24–1.74)
≤3	ref			ref	ref
Relationship status					
Single	0.93 (0.63–1.37)			1.15 (0.68–1.94)	1.07 (0.59–1.95)
Short-term	1.24 (0.59–2.59)			1.53 (0.59–3.98)	1.28 (0.41–4.04)
Stable and long	ref			ref	ref
(**b**)					
	**Model 1**	**Model 2**	**Model 3**	**Model 4**	Model 5 *
	**Risk Ratio (95% CI)**				
Age group (years)					
≥37	1.05 (0.65–1.68)	1.07 (0.64–1.77)			1.01 (0.40–2.30)
<37	ref	ref			ref
Sex					
Male	1.39 (0.86–2.25)	1.22 (0.72–2.08)			1.25 (0.50–3.11)
Female	ref	ref			ref
Education level					
High school or less	0.90 (0.56–1.46)	0.89 (0.52–1.55)			0.88 (0.40–2.01)
Trade or over	ref	ref			ref
Income					
Centrelink	0.87 (0.52–1.46)	1.22 (0.55–2.73)			0.76 (0.25–2.30)
Job	ref	ref			ref
Health care card					
Yes	0.72 (0.42–1.23)	0.62 (0.29–1.31)			0.85 (0.28–2.55)
No	ref	ref			ref
Car ownership					
No	1.21 (0.75–1.96)	1.30 (0.74–2.29)			0.69 (0.27–1.75)
Yes	ref	ref			ref
Smoking status					
Current smoker	1.57 (0.84–2.94)		1.89 (0.94–3.81)		**2.60 (1.05–7.20)**
Ex-smoker	**1.95 (1.01–4.21)**		**1.95 (1.02–4.57)**		**2.90 (1.02–8.60)**
Never smoked	ref		ref		ref
Recreational drug use					
Current user	0.72 (0.35–1.46)		0.67 (0.31–1.43)		0.87 (0.30–2.53)
Ex-user	1.14 (0.68–1.90)		0.82 (0.46–1.48)		0.71 (0.30–1.83)
Never used	ref		ref		ref
Self-rated general health					
Fair/Poor	0.95 (0.56–1.62)		0.75 (0.39–1.43)		0.70 (0.27–1.83)
Excellent/very good/Good	ref		ref		ref
Self-rated oral health					
Fair/Poor	1.08 (0.66–1.79)		1.19 (0.65–2.18)		1.14 (0.50–2.62)
Excellent/very good/Good	ref		ref		ref
Had HPV infection					
Yes	1.44 (0.35–5.92)		1.31 (0.31–5.55)		1.18 (0.13–10.92)
Don’t know	0.85 90.40–1.77)		0.94 (0.43–2.05)		1.57 (0.61–3.98)
No			ref		ref
Ever received HPV vaccination					
No	0.70 (0.33–1.50)		0.72 (0.32–1.60)		0.59 (0.19–1.82)
Don’t know	0.52 (0.23–1.18)		0.48 (0.20–1.19)		**0.30 (0.10–0.95)**
Yes	ref		ref		ref
Ever given oral sex					
Yes	0.78 (0.43–1.39)			-	-
No	ref			-	-
Age first given oral sex (Years)					
<16	1.08 (0.53–2.20)			0.76 (0.33–1.76)	1.17 (0.35–3.46)
≥16	ref			ref	ref
No. of times oral sex given					
>3	0.98 (0.54–1.79)			0.76 (0.33–1.76)	0.48 (0.17–1.34)
≤3	ref			ref	ref
Ever received oral sex					
Yes	0.80 (0.43–1.49)			-	-
No	ref			-	-
Age first received oral sex (Years)					
<16	1.31 (0.71–2.44)			1.52 (0.59–3.92)	1.10 (0.35–3.46)
≥16	ref			ref	ref
No. of times oral sex received					
>3	1.21 (0.67–2.22)			1.36 (0.56–3.29)	1.34 (0.53–3.38)
≤3	ref			ref	ref
Relationship status					
Single	0.95 (0.56–1.60)			0.74 (0.38–1.43)	0.44 (0.05–3.85)
Short-term	0.87 (0.21–3.64)			0.41 (0.06–3.08)	0.35 (0.04–2.19)
Stable and long	ref			ref	ref

Notes: Model 1: crude model; Model 2: the sociodemographic factors model; Model 3: health and health-related behaviours model; Model 4: sexual behaviours model; Model 5: full model. Bold were donated as statistically significant differences. * Using modified Poisson regression with robust standard errors. “-”: RR estimates could not be obtained due to sparse data, or model non-convergence, as applicable.

**Table 3:** (**a**) Multivariable association between persistence of Oral HPV detection and risk factors among non-metropolitan Indigenous Australian adults (n = 482). (**b**) Multivariable association between persistence of Oral HPV detection and risk factors among metropolitan Indigenous Australian adults (n = 298).

(**a**)					
	**Model 1**	**Model 2**	**Model 3**	**Model 4**	**Model 5**
	**Risk Ratio (95% CI)**				
Age group (years)					
≥37	1.20 (0.79–1.84)	1.24 (0.80–1.92)			**2.79 (1.11–7.00)**
<37	ref	ref			ref
Sex					
Male	0.71 (0.44–1.17)	0.72 (0.44–1.19)			0.53 (0.19–1.46)
Female	ref	ref			ref
Education level					
High school or less	1.08 (0.68–1.71)	1.07 (0.66–1.73)			0.97 (0.44–2.14)
Trade or over	ref	ref			ref
Income					
Centrelink	1.16 (0.70–1.92)	1.22 (0.60–2.50)			1.06 (0.30–3.75)
Job	ref	ref			ref
Health care card					
Yes	1.02 (0.60–1.73)	0.89 (0.44–1.79)			1.54 (0.41–5.73)
No	ref	ref			ref
Car ownership					
No	1.09 (0.72–1.67)	1.04 (0.66–1.64)			0.87 (0.36–2.09)
Yes	ref	ref			ref
Smoking status					
Current smoker	0.97 (0.60–1.56)		1.12 (0.67–1.88)		1.00 (0.40–2.53)
Ex-smoker	0.76 (0.34–1.69)		0.81 (0.35–1.90)		0.60 (0.15–2.49)
Never smoked	ref		ref		ref
Recreational drug use					
Current user	0.92 (0.54–1.57)		0.87 (0.48–1.57)		0.96 (0.35–2.64)
Ex-user	0.61 (0.36–1.03)		**0.56 (0.32–0.98)**		0.39 (0.14–1.06)
Never used	ref		ref		ref
Self-rated general health					
Fair/Poor	0.65 (0.35–1.23)		0.68 (0.34–1.33)		0.28 (0.08–1.02)
Excellent/very good/Good	ref		ref		ref
Self-rated oral health					
Fair/Poor	0.88 (0.55–1.39)		1.02 (0.62–1.68)		1.21 (0.54–2.67)
Excellent/very good/Good	ref		ref		ref
Had HPV infection					
Yes	1.36 (0.43–4.34)		1.47 (0.35–6.17)		1.12 (0.23–5.40)
Don’t know	1.35 (0.82–2.20)		1.77 (1.01–3.09)		1.33 (0.49–3.60)
No	ref		ref		ref
Ever received HPV vaccination					
No	2.05 (0.74–5.66)		1.99 (0.71–5.59)		0.49 (0.12–1.90)
Don’t know	1.63 (0.57–4.65)		1.46 (0.49–4.34)		0.62 (0.16–2.40)
Yes	ref		ref		ref
Ever given oral sex					
Yes	0.93 (0.59–1.48)			-	-
No	ref			-	-
Age first given oral sex (Years)					
<16	1.18 (0.59–2.33)			2.06 (0.79–5.35)	1.70 (0.62–4.62)
≥ 16	ref			ref	ref
No. of times oral sex given					
>3	0.91 (0.49–1.69)			0.98 (0.37–2.59)	0.83 (0.22–3.12)
≤3	ref			ref	ref
Ever received oral sex					
Yes	0.69 (0.44–1.10)			-	-
No	ref			-	-
Age first received oral sex (Years)					
<16	0.64 (0.27–1.52)			0.39 (0.13–1.17)	0.52 (0.17–1.64)
≥ 16	ref			ref	ref
No. of times oral sex received					
>3	1.23 (0.64–2.34)			1.45 (0.55–3.83)	2.70 (0.67–10.83)
≤3	ref			ref	ref
Relationship status					
Single	1.09 (0.68–1.73)			0.50 (0.07–3.75)	0.46 (0.05–4.00)
Short-term	0.99 (0.35–2.76)			0.87 (0.43–1.76)	1.02 (0.46–2.23)
Stable and long	Ref			ref	ref
(**b**)					
	**Model 1**	**Model 2**	**Model 3**	**Model 4**	**Model 5**
	**Risk Ratio (95% CI)**				
Age group (years)					
≥37	1.02 (0.52–1.97)	1.12 (0.56–2.22)			1.12 (0.45–2.83)
<37	ref	ref			ref
Sex					
Male	1.21 (0.61–2.39)	1.17 (0.56–2.44)			1.09 (0.40–3.01)
Female	ref	ref			ref
Education level					
High school or less	1.68 (0.81–3.50)	1.50 (0.68–3.32)			2.42 (0.78–7.49)
Trade or over	ref	ref			ref
Income					
Centrelink	1.35 (0.61–2.96)	0.85 (0.28–2.58)			1.46 (0.41–5.22)
Job	ref	ref			ref
Health care card					
Yes	1.57 (0.65–3.79)	1.56 (0.49–4.96)			1.25 (0.30–5.26)
No	ref	Ref			ref
Car ownership					
No	1.30 (0.66–2.54)	1.08 (0.52–2.27)			0.50 (0.15–1.69)
Yes	ref	ref			ref
Smoking status					
Current smoker	0.85 (0.40–1.81)		0.61 (0.25–1.46)		0.54 (0.16–1.88)
Ex-smoker	0.71 (0.23–2.23)		0.49 (0.13–1.84)		0.17 (0.03–1.12)
Never smoked	ref		ref		ref
Recreational drug use					
Current user	1.71 (0.75–3.89)		2.17 (0.78–5.99)		0.99 (0.28–3.51)
Ex-user	1.20 (0.55–2.64)		1.36 (0.55–3.35)		0.83 (0.26–2.66)
Never used	ref		ref		ref
Self-rated general health					
Fair/Poor	0.98 (0.47–2.05)		1.10 (0.47–2.60)		2.16 (0.73–6.37)
Excellent/very good/Good	ref		ref		ref
Self-rated oral health					
Fair/Poor	1.21 (0.61–2.40)		0.92 (0.40–2.08)		0.62 (0.22–1.76)
Excellent/very good/Good	ref		ref		ref
Had HPV infection					
Yes	1.33 (0.18–9.73)		1.13 (0.15–8.81)		1.45 (0.13–15.59)
Don’t know	0.39 (0.09–1.62)		0.44 (0.10–1.92)		0.31 (0.04–2.49)
No	ref		ref		ref
Ever received HPV vaccination					
No	1.12 (0.34–3.73)		1.10 (0.32–3.81)		0.94 (0.17–5.27)
Don’t know	0.51 (0.13–1.96)		0.54 (0.13–2.18)		0.39 (0.06–2.37)
Yes	ref		ref		ref
Ever given oral sex					
Yes	1.49 (0.56–3.88)			-	-
No	ref			-	-
Age first given oral sex (Years)					
<16	**2.63 (1.19–5.79)**			**2.72 (1.06–10.46)**	**2.82 (1.08–11.73)**
≥16	ref			ref	ref
No. of times oral sex given					
>3	1.47 (0.66–3.27)			0.91 (0.28–2.90)	1.62 (0.45–5.83)
≤3	ref			ref	ref
Ever received oral sex					
Yes	**3.15 (1.00–13.3)**			-	-
No	ref			-	-
Age first received oral sex (Years)					
<16	1.90 (0.88–4.11)			0.84 (0.21–3.37)	1.53 (0.33–7.10)
≥16	ref			ref	ref
No. of times oral sex received					
>3	1.54 (0.70–3.39)			1.31 (0.38–4.51)	1.06 (0.28–3.96)
≤3	ref			ref	ref
Relationship status					
Single	1.26 (0.61–2.61)			1.19 (0.52–2.73)	1.11 (0.42–2.96)
Short-term	1.93 (0.44–8.49)			1.96 (0.43–8.89)	1.29 (0.23–6.93)
Stable and long	ref			ref	ref

Notes: Model 1: crude model; Model 2: the sociodemographic factors model; Model 3: health and health-related behaviours model; Model 4: sexual behaviours model; Model 5: full model. Bold were donated as statistically significant differences.

## Data Availability

The datasets generated and/or analyzed during the current study are not publicly available due to privacy issues of the participants. Data is available from the corresponding author on reasonable request.

## Supplementary Materials

The following supporting information can be downloaded at https://www.mdpi.com/article/10.3390/v18101054/s1, Figure S1: Flow chart of data collection at baseline (2018–19), 12 and 24 months among Indigenous Australian adults [18].

## Abbreviations

The following abbreviations are used in this manuscript:

ACCHOs	Aboriginal Community Controlled Health Organisations
ATSI	Aboriginal and Torres Strait Islander
HPV	Human papillomavirus
hrHPV	high-risk HPV
lrHPV	low-risk HPV
OPC	Oropharyngeal cancer
