# Supplementary material for: Assessing the Incidence, Persistence and Clearance of Oral HPV Detection Among Metropolitan and Non-Metropolitan Indigenous Australian Adults
Source: Viruses. 2026 Sep 23;18(10):1054. doi: 10.3390/v18101054 (PMC13709348; doi:10.3390/v18101054)
Supplement: Supplementary file 1 [file viruses-18-01054-s001.zip › viruses-4504159-supplementary.pdf]

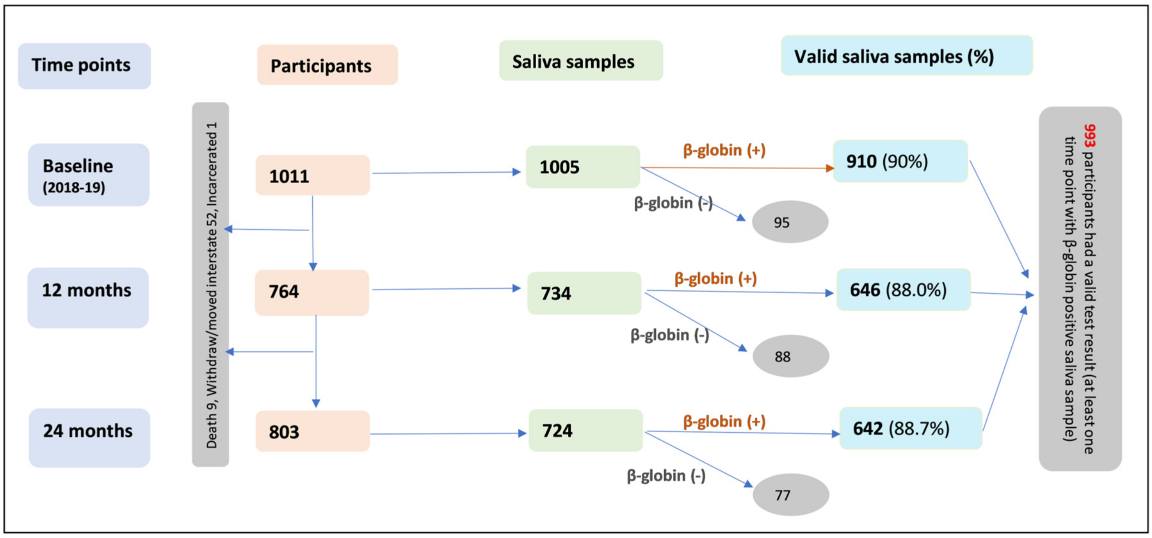

Figure S1: Flow chart of data collection at baseline (2018–19), 12 and 24 months among Indigenous Australian adults [18]

[18] Ju, X.; Sethi, S.; Antonsson, A.; Hedges, J.; Canfell, K.; Smith, M.; Garvey, G.; Logan, R.M.; Jamieson, L.M. Natural History of Oral HPV Infection among Indigenous South Australians. Viruses 2023, 15, 1573.
